# Deep Learning Multi-Class Approach for Human Fall Detection Based on Doppler Signatures

**DOI:** 10.3390/ijerph20021123

**Published:** 2023-01-08

**Authors:** Jorge D. Cardenas, Carlos A. Gutierrez, Ruth Aguilar-Ponce

**Affiliations:** Facultad de Ciencias, Universidad Autónoma de San Luis Potosí, Av. Chapultepec 1570, Privadas del Pedregal, San Luis Potosí C.P. 78295, Mexico

**Keywords:** fall detection, WiFi, LSTM, CNN, elderly healthcare, Doppler signatures

## Abstract

Falling events are a global health concern with short- and long-term physical and psychological implications, especially for the elderly population. This work aims to monitor human activity in an indoor environment and recognize falling events without requiring users to carry a device or sensor on their bodies. A sensing platform based on the transmission of a continuous wave (CW) radio-frequency (RF) probe signal was developed using general-purpose equipment. The CW probe signal is similar to the pilot subcarriers transmitted by commercial off-the-shelf WiFi devices. As a result, our methodology can easily be integrated into a joint radio sensing and communication scheme. The sensing process is carried out by analyzing the changes in phase, amplitude, and frequency that the probe signal suffers when it is reflected or scattered by static and moving bodies. These features are commonly extracted from the channel state information (CSI) of WiFi signals. However, CSI relies on complex data acquisition and channel estimation processes. Doppler radars have also been used to monitor human activity. While effective, a radar-based fall detection system requires dedicated hardware. In this paper, we follow an alternative method to characterize falling events on the basis of the Doppler signatures imprinted on the CW probe signal by a falling person. A multi-class deep learning framework for classification was conceived to differentiate falling events from other activities that can be performed in indoor environments. Two neural network models were implemented. The first is based on a long-short-term memory network (LSTM) and the second on a convolutional neural network (CNN). A series of experiments comprising 11 subjects were conducted to collect empirical data and test the system’s performance. Falls were detected with an accuracy of 92.1% for the LSTM case, while for the CNN, an accuracy rate of 92.1% was obtained. The results demonstrate the viability of human fall detection based on a radio sensing system such as the one described in this paper.

## 1. Introduction

According to the World Health Organization, in 2018, it was reported for the first time in history that the number of people over 65 exceeded the number of people under 5 years of age worldwide [1]. Therefore, it is estimated that by 2050, one in six people will belong to the elderly sector [2]. The fact that elderly people live alone raises concerns because they might not have access to continuous health status monitoring. Falls are the second leading cause of death worldwide, causing injuries that require immediate medical attention; otherwise, they can be fatal [3]. Unfortunately, the rate of deaths caused by falls continues to increase each year, and if this growth continues, it is anticipated that by 2030, there will be 7 fatalities every hour [4]. In addition, several studies show that if falls are not fatal, there is a possibility that people present physical and psychological complications for the rest of their lives [5]. Some physical complications include restrictions in daily activities, joint pain, broken bones, or brain injuries, among others. Falls are the most common mechanism for causing traumatic brain injuries [6]. Psychologically, the person struggles with sadness, a lack of self-assurance, and a fear of falling again. On the other hand, statistics show that 80% of fall deaths are in low- and middle-income countries [3]. For this reason, the development of low-cost fall detection systems is a critical need.

This work aims at detecting human falls through radio sensing based on a continuous wave (CW) radio-frequency (RF) probe signal that can be transmitted as a pilot signal within the communications signal frame. Pilot signals do not carry information data and are used only for synchronization purposes between the transmitter and the receiver [7]. However, these signals are subject to frequency dispersions caused by the Doppler effect. Such frequency dispersions are known as Doppler signatures and have been widely used for sensing purposes [8,9,10]. The Doppler signatures produced by the interaction of the CW probe signal with a moving person can be analyzed to characterize falling events. We developed a CW probe signal transmission and reception system using general-purpose equipment that can be easily replicated. Unlike other works where a single-input multiple-output scheme is followed [11,12,13], our system only requires a single-input single-output (SISO) radio link. Furthermore, the platform allows the acquisition of the information from the probe signals through a less complex process than channel state information (CSI) or received signal strength (RSSI) estimation.

In a previous study [14], we presented the influence of antenna orientation in fall detection systems using a SISO RF signal transmission platform. The results determined the orientation of the antennas with which the Doppler signatures of a fall are captured with the best resolution. However, this proof of concept was performed using a binary classification of events (falls and no activity). As a significant extension of this work, we seek to increase the robustness of the system using a multi-class classification approach and increase accuracy by implementing advanced deep learning (DL) techniques. DL is considered one of the core technologies of artificial intelligence capable of building intelligent systems [15]. Machine learning algorithms can be classified as bag-of-feature (BoF) or model algorithms [16]. BoF performs classification by a) extracting desirable features, b) generating a codebook, and c) generating a vector of features. The main problem in BoF is feature selection that guarantees separability between classes. Features must exhibit high information-packing properties, a high discrimination rate between classes, low within-class variability, and the ability to discard redundant information. Model-based machine learning, such as DL, performs feature extraction and classification within the designed network. The training process establishes which features are relevant to the desired classification. DL automatically selects the feature that achieves the best classification. In our methodology, the model will extract the required features from the received CW probe signals for the learning process. The system is trained to identify the Doppler signatures of different activities of daily life and specific events such as walking, going up/down stairs, and falling. We implement and evaluate two one-dimensional neural network algorithms for this process: a long-short-term memory network (LSTM) and a convolutional neural network (CNN). The LSTM network is considered one of the most successful recurrent neural networks [15]. Our implementation achieved 92.1% accuracy in detecting falling events with the LSTM algorithm. On the other hand, CNN is a popular feature discriminator that automatically learns from the input signal without a feature extraction pre-process [17]. We assessed the CNN classification algorithm, and we achieved an accuracy of 92.1% on falling events. Our results shows that the Doppler signatures of a CW probe signal are sufficient for accurately detecting falling events and differentiating them from other activities.

The rest of the paper is organized as follows. Section 2 provides a review of related work. Section 3 shows the materials and methods used for sensing and capturing the Doppler signatures from human activities. Section 4 describes the architectures of the implemented classification framework. Section 5 addresses the results and discussion of the proposed framework and its performance. Finally, Section 6 presents the conclusions.

## 2. Related Work

Several systems have been proposed for fall detection using different sensing techniques. A machine vision technique can be implemented for this purpose. These systems are based on digital image processing and need to capture high-resolution images to achieve a high accuracy rate [18]. However, not all vision systems can deliver high image quality, and investing in equipment with higher resolution is an expensive solution. Furthermore, partial or complete occlusion of people by objects or walls reduces the detection of events [19]. Environment-based systems have the same disadvantages due to requiring the installation of acoustic or vibration sensors throughout the entire indoor environment [20,21]. Due to their sophisticated implementations and high cost, they are not always viable. On the other hand, wearable devices have taken advantage of low-cost sensors embedded in general-purpose devices that people can wear to be monitored [22,23,24]. Nevertheless, the subject’s will to wear such sensing devices is of prime importance because detection accuracy depends on it. However, users might find it uncomfortable to keep the device attached to their bodies. This approach is invasive and must be worn at all times in order to detect falls accurately.

An innovative approach to fall detection is through a RF technique. The operation of these systems is based on the transmission of RF signals that are reflected, diffracted, and scattered by static or moving human bodies [11]. Changes in such signals resulting from the interaction with the human body can thus be characterized to determine the nature of the movement performed. This means that the RF systems can monitor activities without the need for the user to wear a device on their body. The construction of these RF-based sensors can be implemented with relatively simple circuits to generate CW signals [25]. In addition, the health data provided by these signals can be monitored through technologies such as IEEE 802.11af, which provides opportunistic access to licensed channels with the help of cognitive functions without any spectrum cost [26]. Therefore, the development of these technologies makes RF-based fall monitoring systems a low-cost alternative.

The RF signal transmitter that has had the greatest relevance in the field of monitoring systems is the WiFi access point (AP) [27]. These devices are ubiquitous in most indoor environments and are a commercial off-the-shelf technology. WiFi signals are commonly analyzed through the CSI [28,29] or the RSSI [30]. The CSI provides information about changes in signal amplitude or phase during a falling event, whereas the RSSI measures changes in the average strength of the signal. In fall detection, CSI analysis has stood out above other approaches and has shown promising results. However, the technology available for the implementation of these systems is still limited principally by laborious offline processing. For example, sensing applications using CSI require a high rate of measurements, which reduces network performance and efficiency [31]. Furthermore, the APs have to work in data transmission mode to compute the necessary orthogonal frequency division multiplexing (OFDM) signal for the CSI, which cannot always be guaranteed [32]. These aspects compromise the coexistence of joint radio sensing and communication systems. Therefore, it is necessary to define a new methodology that integrates both schemes.

Radar devices are another well-established sensing technology that is robust to the environment, small in size, and which has operating frequencies ranging from a few GHz to hundreds of GHz [33]. Doppler radars have been widely used to monitor human activity. Unfortunately, these systems require specialized hardware, which can be expensive to develop. Furthermore, signal processing for radar sensing requires information about the cross-correlation between surveillance and reference signals that are not part of the communications signal frame [34].

## 3. Materials and Methods

### 3.1. System Overview

**Figure 1:**
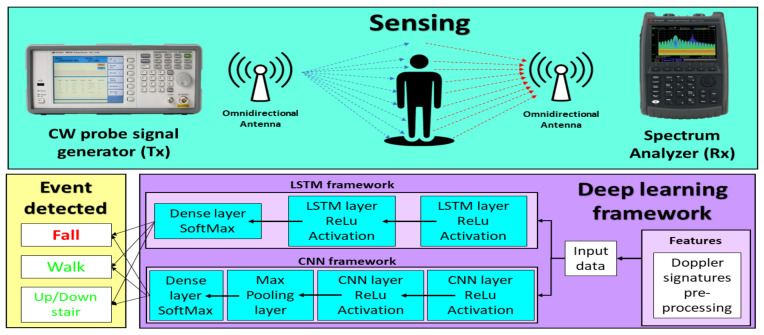
Overview of the fall detection system based on Doppler signatures.

Figure 1 shows an overview of the implemented platform for fall detection based on CW probe signals. In the experimental stage, the sensing of human movements is carried out through the transmission of RF probe signals in an indoor environment. Some of the signals are absorbed by the human body, and others are reflected or scattered toward the receiver. Therefore, the reception of the signal occurs through more than one path and at different times. This is known as multipath propagation. The Doppler effect causes frequency shifts in RF signals and occurs when a moving body interacts with the transmitted signal. A Doppler signature is the frequency dispersion pattern imprinted on the probe signal during an activity or event. We intend to associate the falling events with the features of their generated Doppler signatures. Our efforts are focused on the implementation of two DL frameworks capable of accurately detecting falling events and distinguishing them from human activities. The LSTM network and CNN have shown remarkable success in performing fall detection tasks [12,23,35].

### 3.2. Sensing Platform

A Keysight N9310A RF signal generator can emit continuous waves from 9 kHz to 3 GHz, with a power of 20 dBm. We use this general-purpose equipment to transmit CW probe signals in an indoor environment. The transmission is carried out with an omnidirectional monopole antenna (Tx) of 9 dBi gain that works in the 2.4–2.5 GHz band. Antennas with these characteristics are commonly used in WiFi. Furthermore, a Keysight FieldFox N9912A (Rx) spectrum analyzer was used to capture the transmitted signals and to characterize the coupling of the antennas with the transmitting and receiving equipment. According to our measurements, the monopole antennas had a better coupling at a frequency of 2.42 GHz. These antennas were fixed with a horizontal orientation for both Tx and Rx. This is in agreement with the results reported in our previous work, where it was shown that this orientation improves the resolution with which the Doppler signatures are recorded [14]. Finally, the sweep time for detection was fixed at 271 ms with a frequency span of 1.5 kHz because the duration of a fall is between 1–1.5 s.

### 3.3. Recruitment of Participants

The experiments in this study were conducted with a total of 11 adult participants. The age of the people involved was in a range between 21 and 48 years of age. Recruitment took place in the city of San Luis Potosí, Mexico, where the volunteers declared that they were in good physical health. Among the requirements to demonstrate good health is not having a history of musculoskeletal problems. The volunteer population was divided into 6 men and 5 women with different body masses and heights (Table 1). This variation in the physiognomy of the participants enables data to be obtained from activities performed at different speed. Furthermore, the amount of Doppler dispersion produced by the volunteers varies according to their weight or height. Before the experiments, the participants filled out informed consent forms about the risks of the study. Furthermore, all the experiments were carried out under the ethical standards that the Autonomous University of San Luis Potosí demands. However, our experiments did not require the approval of an ethics committee as they did not involve the use of pharmaceuticals, genomic studies, the participation of health professionals, or collaboration with any medical institution. Therefore, we followed the protocols of the Declaration of Helsinki and the participants signed the informed consent generated for this research work.

### 3.4. Experimental Protocol

**Figure 2:**
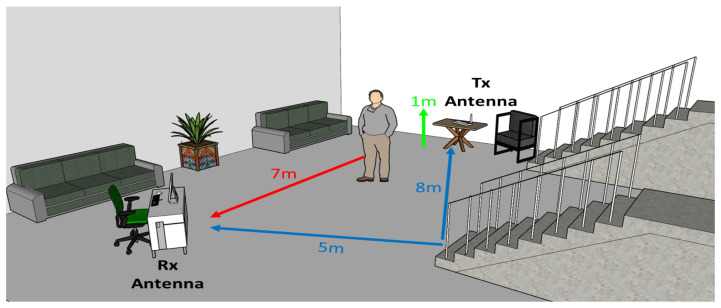
Test scenario for fall detection experiments and positioning of the measurement equipment.

The sensing platform was located within the facilities of the Autonomous University of San Luis Potosí, Mexico. Each experiment was carried out in an indoor environment that simulates a two-story home. Figure 2 shows the dimensions of the test scenario and the position of the measurement equipment. The experiments were divided into 4 classes of activities to be carried out by the participants. The first activity consisted of a constant walk of 7m distance in a straight direction. Each participant performed 30 repetitions, changing the direction in which they moved to cover the entire perimeter of the indoor environment. The second activity was performed by going up and down a 10-step ladder for a total of ten repetitions per participant. Finally, the volunteers had to simulate a fall situation both in the case of fainting and a fall resulting from going down the stairs. For this, a thin mattress and an airbed were placed in the room to reduce the impact force caused by falls. In the scenario of a fainting event, the person was placed in the center of the room to perform the movement. The event was replicated 5 times per participant. On the other hand, in the event of a fall from the stairs, the participant stood 2 steps up and descended the stairs, ending with a falling movement towards the padded surface. Five repetitions of this event were performed. Furthermore, we included a series of experiments that did not require the presence of the participants. These experiments consisted of taking snapshots of the probe signal in the indoor environment without any human movement involved. The data collected in this experiment were categorized into a particular class of “no activity” and were used to determine when a person remains static. The summary of the activities and repetitions developed by the participants is shown in Table 2.

### 3.5. Doppler Signatures Analysis

A methodology that allows visualizing the changes in the spectral density in a short observation time is necessary to differentiate the Doppler signatures recorded in the CW signals during the performance of activities. This has the objective of determining if the features of each signature are unique according to the activity to which they correspond. The spectrogram method is used to analyze time-varying and non-stationary signals, such as those transmitted by our detection system. Therefore, we propose to compute spectrograms for the generation of a database containing the sequence of activities recorded by the platform.

**Figure 3:**
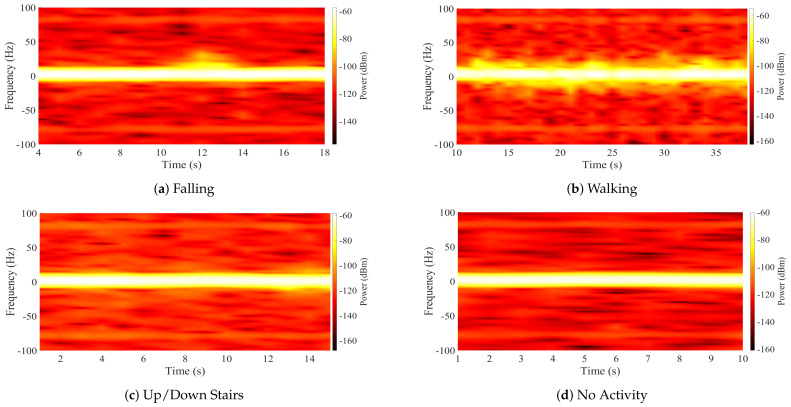
Spectrograms of the four activities performed in the indoor environment.

The spectrogram of a received CW RF signal is calculated as $S\left(t^{\prime };\nu \right)={\left|Y\left(t^{\prime };\nu \right)\right|}^{2}$, where $t^{\prime }$ is the observation time, $Y\left(t^{\prime };\nu \right)=\int_{-\infty }^{\infty }y\left(t\right)w\left(t-t^{\prime }\right)e^{-j2\pi \nu t}\;dt$ is the Fourier transform of y(t), and w(t) is a positive window and energy unit pair [36]. The shape and length of the window function w(t) must be optimized to obtain a good resolution in frequency and a reduced spectral loss. A Gaussian window fits the time-frequency analysis requirements of the application at hand. Thereby, w(t) can be given by

(1) $$\begin{array}{c} w\left(t\right)={\left(\sqrt{\pi }\sigma_{s}\right)}^{-1/2}e^{-t^{2}/\left(2\sigma_{s}^{2}\right),} \end{array}$$

where $\sigma_{s}$ is the dispersion parameter [37]. Examples of the spectrograms recorded during the four activities considered in our experiments are shown in Figure 3.

#### 3.5.1. Doppler Signature Pre-Processing

**Figure 4:**
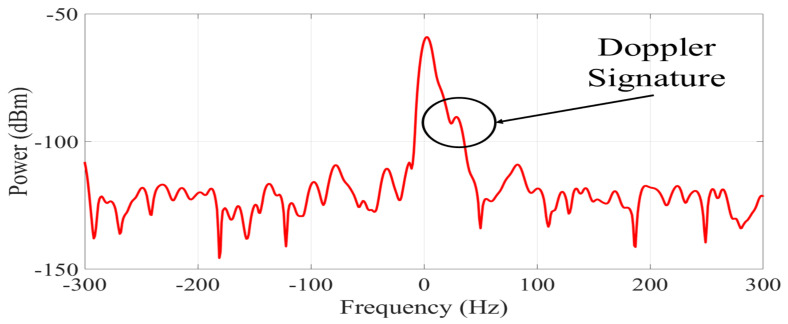
Snapshot of the probe signal during a falling event without applying a pre-processing stage.

**Figure 5:**
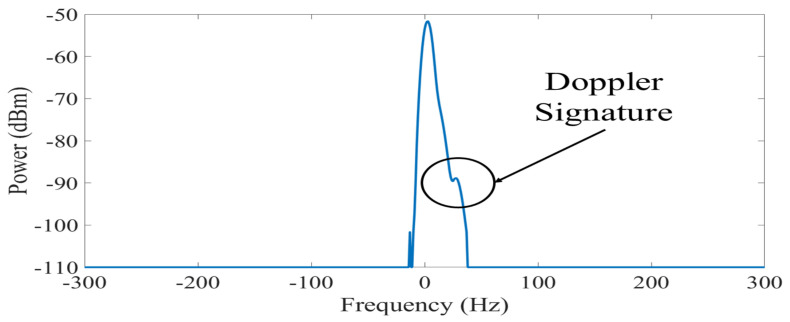
Probe signal pre-processed with denoising filter.

**Figure 6:**
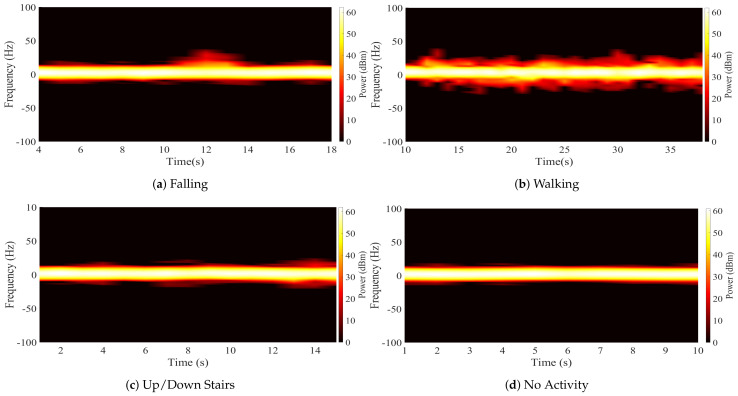
Spectrograms of the activities performed during the experimentation protocol after noise removal.

The RF signal received by the platform contains an additive white Gaussian noise component $n_{\sigma }\left(t\right)\in C$; C refers to the set of complex-valued numbers [14]. This component corrupts the signal mainly due to thermal noise from the measurement equipment and affects the resolution with which the signals are captured. Therefore, it can be challenging to extract and analyze the Doppler signatures. The noise contribution in the received signal is illustrated in Figure 4, which is a capture of the spectrogram of Figure 3a at a time $t^{\prime }=12$ s. We observe that the RF signal dispersion caused by a falling person can be masked by noise. It is required to construct a pre-processing stage that enables us to sanitize the RF signals and remove the majority of the noise in order to account for these practical concerns. To compensate for these practical issues, it is necessary to implement a pre-processing stage. We propose a pre-processing methodology based on a denoising filter through a Gaussian mask. The masking is done through a Gaussian signal with an amplitude that properly covers the bandwidth of the sub-carrier signal and the Doppler generated by a person during a falling event. The mask was obtained by averaging the snapshots of the spectrograms captured during the experiments and was applied to the original values of the received signals. Figure 5 shows the result of applying the Gaussian mask to the spectrum of Figure 4, where the noise was smoothed in most of the signal. Furthermore, it is shown that the dispersion generated by the Doppler effect of the fall is preserved and is simpler to visualize. Figure 6 shows the calculated spectrograms after sanitizing the RF signals in all activities. These spectrograms are used to build the database for our fall detection platform. However, the sequences of activities do not have the same number of samples, and it was necessary to take a fixed observation window. The observation window was set at 10 samples per spectrogram because the dispersion of falls does not exceed 3 samples and is sufficient to capture a sequence of walking and going up or down stairs. According to Table 2, for a total of 660 calculated spectrograms, a database with 6600 samples was generated. Furthermore, after the probe signals are pre-processed, most of the information in the frequency spectrums is negligible (Figure 5). In our case, the maximum values of the power of the probe signal and its Doppler signatures were preserved in 82 values of the snapshots. These values are considered the principal features of the experiments.

## 4. Deep Learning Framework

DL is a methodology that has been proposed to perform classification and regression tasks with significant results in the area of data science [15,38]. DL algorithms are based on artificial neural networks to build computational models capable of extracting and learning the principal features of a data set. The first step in developing a DL model is to create a data set that includes measurements of various activities. The dispersion caused by the acceleration of falling bodies has a progressive and natural sequence. Moreover, human activities such as walking and going up or down stairs are also sequential activities. In order to analyze those activities, a sequence of spectrums conforming to an activity must be formed. A window that included ten spectrums allowed us to provide sequences where the activities could be observed. Each activity is measured with a sequence of spectrograms. Our data set was built with a set of different experiments involving a predetermined set of activities. According to Table 2, a total of 660 sequences were captured and divided into 4 different classes. For the classification process, the algorithms need one data set for training and another for validation and testing. Our training data corresponds to 80% of the total captured sequences, and the remaining 20% was used for validation and testing.

In this work, we consider two architectures with different supervised learning approaches to compare the features they are capable of extracting and determine which have better classification results. The first architecture is the LSTM network, which has a generative approach, and, on the other hand, the second architecture is a CNN that has a discriminative approach [39]. These two architectures are described in detail below.

### 4.1. Long Short-Term Memory Network

LSTM networks are a variation of recurrent neural networks and are used in sequential data analysis applications [9,40]. These networks have a state cell, which is a memory block that stores data for long periods. This solves the vanishing gradients problem, which makes it difficult for the network to remember important information from the beginning of the sequence as the sequence increases. The memory block is divided into three gates: the forget gate, the input gate, and the output gate. The forget gate has the task of determining what information from the previous state cell will be stored and what information can be discarded. On the other hand, the input gate decides the data to enter the state cell and the output gate controls the outputs. Therefore, we can use a larger volume of input data to increase the efficiency of network learning without losing information over long periods.

**Figure 7:**
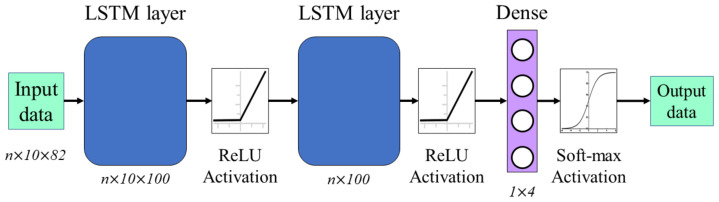
The architecture of the LSTM network implemented for the classification process, where *n* is the size of the input data set.

The architecture of our classification algorithm using LSTM networks is shown in Figure 7. The input data in these networks must be a three-dimensional array. The first dimension is the one that represents the batch size, the second dimension corresponds to the time steps, and finally, the third dimension is the number of units or features in an input sequence. The batch size was 5391 samples for the training set and 1191 samples for the validation and test set, which correspond to 80% and 20% of the total samples of the database, respectively. The time step taken has a value of 10, considering the size of the observation window of the pre-processing stage. The features entered into the network were the 82 most relevant values of the probe signals (Section 3.5.1). Once the data were entered into the algorithm, they were processed by two LSTM network layers using a rectified linear unit (ReLU) activation function. The number of input artificial neurons in both layers was 100. Finally, the output is a dense layer that classifies the samples into the 4 given activity classes using a softmax activation function.

### 4.2. Convolutional Neural Network

**Figure 8:**
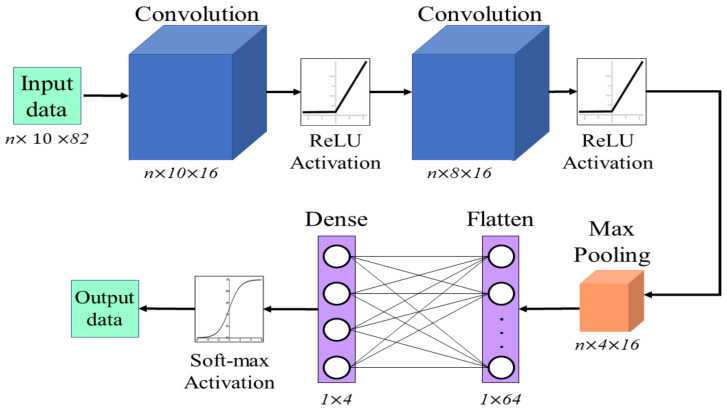
The architecture of the CNN network implemented for the classification process, where *n* is the size of the input data set.

CNNs are classification models that can reduce the dimensionality of the input data and extract the principal features without requiring a supervised extraction stage. This architecture is mainly based on the implementation of convolution layers to discriminate a data set. The convolution is computed through multiple digital filters that sweep the input data and create a feature map. Pooling layers are used for dimensionality and operation reduction as well as feature summarization. A max pooling layer takes only the maximum value of a portion of the feature map given by a defined kernel and discards the rest. This process reduces the computational cost of algorithms by extracting and preserving only the dominant features of the data set. Finally, a fully connected layer called a dense layer is used to determine the class to which each extracted feature belongs. For this, it is first necessary that a flatten layer must process the data extracted from the convolution layer to adjust its dimensions to those of the dense layer. Therefore, the proposed CNN architecture for fall detection is defined in Figure 8. The input data of the algorithm have to be a three-dimensional array represented by the batch size, the time step, and the number of features. In our implementation, 16 filters were used in each of the convolution layers. The stride, which defines the input of the given data, was set to 1 for all layers. We selected a kernel value of 3 to reduce the computational cost without losing excessive details on the features. The convolution layers were built using a ReLU activation function. Finally, for the fully connected layer, a softmax activation was selected for the dense layer.

### 4.3. Performance Evaluation

We use the confusion matrix to visualize the results and determine the percentage of activities classified correctly. In a confusion matrix, 4 possible results can be obtained: true positives (TP), true negatives (TN), false negatives (FN), and false positives (FP). Positive true values are the number of samples that were correctly classified in the class being evaluated. The true negatives are the samples correctly classified in the rest of the classes. On the other hand, the false negatives are the samples of the evaluated class that were classified as another activity. Finally, the false positives, contrary to the false negatives, are the samples of other activities that were classified as the evaluated class. To evaluate the classification performance of the classes from the confusion matrices, some metrics can be used, including accuracy, precision, recall, and specificity. These metrics are computed using the following equations:

(2) $$Accuracy=\frac{TP+TN}{TP+FP+FN+TN}$$

(3) $$Precision=\frac{TP}{TP+FP}$$

(4) $$Recall=\frac{TP}{TP+FN}$$

(5) $$Specificity=\frac{TN}{TN+FP}$$

where accuracy refers to how close the result is to its true value, precision is how close the results are to each other, recall is the proportion of TP that were correctly classified, and specificity is the rate of TN which expresses how well the model can detect a class. The set of results of Equations (2)–(5) are in a normalized range between 0 and 1, corresponding to 0% and 100%, respectively. Furthermore, based on the performance evaluation by confusion matrices, the receiver-operating characteristic (ROC) curve can be computed. This is a graph used to visualize the performance of the model, and it represents two parameters: sensitivity and specificity. The area under the ROC curve (AUC) measures the two-dimensional area under the ROC and provides an aggregate measure of performance. The AUC measures how well the predictions of each class are classified and their quality. AUC values range from 0 to 1, where 0 represents that all predictions were incorrect, and 1 represents that they were all correct.

## 5. Results and Discussion

**Figure 9:**
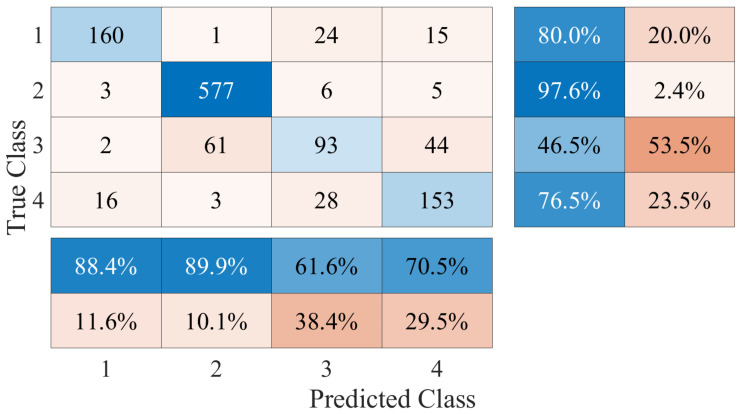
LSTM multi-class classification confusion matrix results, where the classes correspond to (1) no activity, (2) walking, (3) going up/down stairs, and (4) falling.

**Figure 10:**
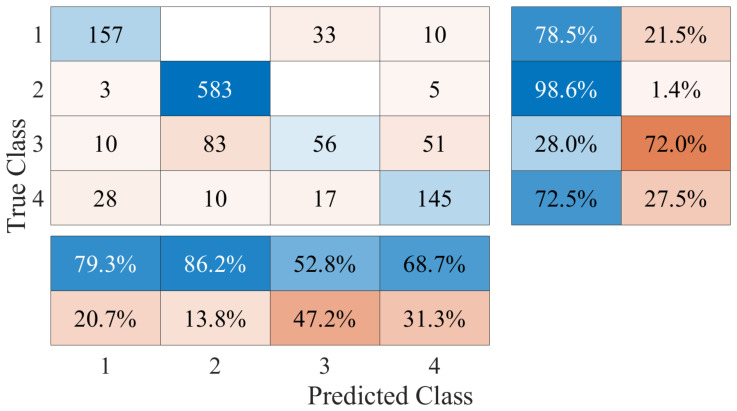
CNN multi-class classification confusion matrix results, where the classes correspond to (1) no activity, (2) walking, (3) going up/down stairs, and (4) falling.

The performance of the fall detection platform based on a DL framework was evaluated by taking the results of the LSTM and CNN classification algorithms. The classification was performed with a multi-class approach, taking 4 different activities from the generated database. The sequence of samples of each activity was assigned a numerical label such that no activity corresponds to class 1, walking corresponds to class 2, going up and down stairs corresponds to class 3, and the falling event is class 4. The confusion matrix generated with the results of the LSTM algorithm is shown in Figure 9. From this confusion matrix, we computed the performance metrics that are listed in Table 3. The accuracy achieved for this framework was 82.20%. On the other hand, Figure 10 shows the confusion matrix generated with the results of the CNN. Considering the values registered in the matrix, the metrics were calculated to evaluate the performance and are shown in Table 4. In this case, the accuracy achieved in the classification of all activities was 78.09%. Comparing the results, the LSTM algorithm obtained the best performance. The performance success rate demonstrates that Doppler signatures can be used as the main feature of classification systems and activity recognition applications. However, the aim of the platform is not to recognize human activities, but to differentiate falls from other classes. Therefore, the class that is required to have the highest possible accuracy rate should be the falling class. In total, 70.50% of the falling events were correctly detected and classified for the LSTM case; this value was 68.70% for the CNN. These results indicate that there is a high rate of falls that cannot be detected or are misclassified. Figure 9 and Figure 10 show how the class corresponding to going up and down stairs generates the highest percentage of FP in the falling class. Therefore, there are a large number of samples of going up and down stairs that are confused with falls. This is due to the resolution of the Doppler signatures captured during the experiments, which is directly related to the measurement equipment. It is clear from examining the spectrogram of someone climbing and descending stairs in Figure 6 that the fluctuations in the spectrum exhibit behavior resembling that of falls but with significantly smaller amplitudes. This behavior implies that during the learning process, the algorithms cannot differentiate the features of these two classes. This directly influences the performance in detecting falling events. Finding a solution to this is central to our classification framework.

**Figure 11:**
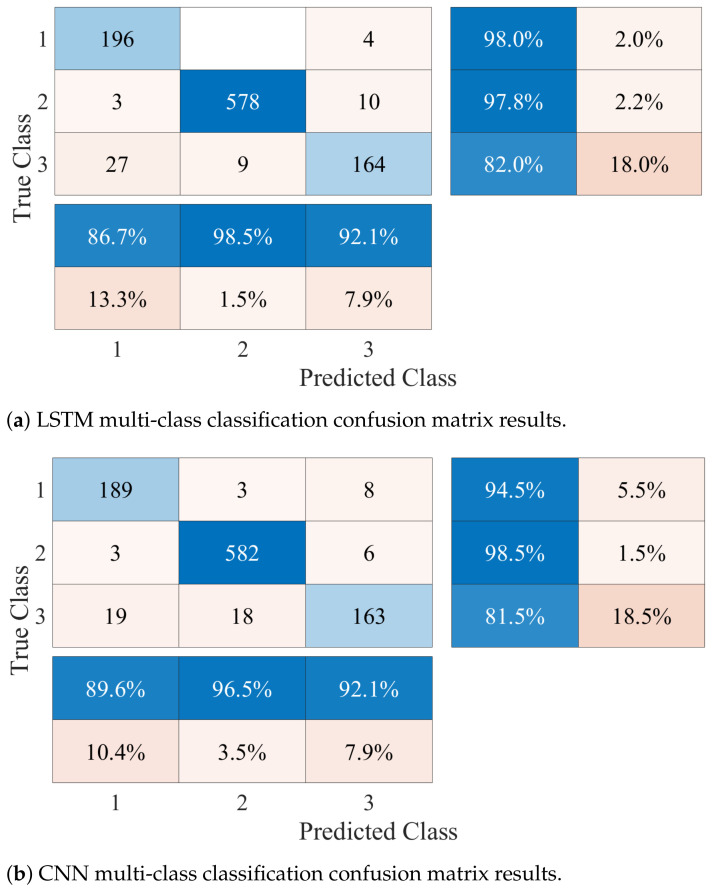
Confusion matrices of both classification frameworks where the classes correspond to (1) no activity, (2) walking, and (3) falling.

There are some techniques that can be used to acquire Doppler signatures with higher resolution during experiments. For instance, improved feature extraction inside the network can be achieved by using better RF signal acquisition resolution. Measurement equipment with higher performance can be used to avoid the loss of power of the signals in long-distance indoor environments. Furthermore, sophisticated pre-processing methods can be used to enhance classification. However, due to the physical difficulties and the risk involved for an elderly person going up and down stairs, this sector of the population has been limited to living in one-story houses. Therefore, we can limit the range of activities in our multi-class approach to only those that generate enough Doppler spread to be captured by the sensing platform. Following the sensing process previously described, the experimental protocol was repeated only by considering the classes of no activity, walking, and falls. In this way, the same DL architectures were used for data classification. The system was tested and analyzed following these metrics, and the results were used to compute the confusion matrices shown in Figure 11. From the confusion matrix in Figure 11a, the accuracy of the LSTM algorithm was calculated and a value of 94.95% was obtained. This result had a considerable increase when compared to the scheme that considers the 4-class classification. The precision in falls also had an improvement with a percentage of 92.1% and a recall of 82.0%. On the other hand, using the CNN architecture, the accuracy achieved was 94.25%, an improvement of more than 16%. Falls reported a precision of 92.1%, with a sensitivity of 81.5%. Notably, the results in both algorithms were very similar; in addition, they reported a false alarm rate with a maximum of 7.90%.

**Figure 12:**
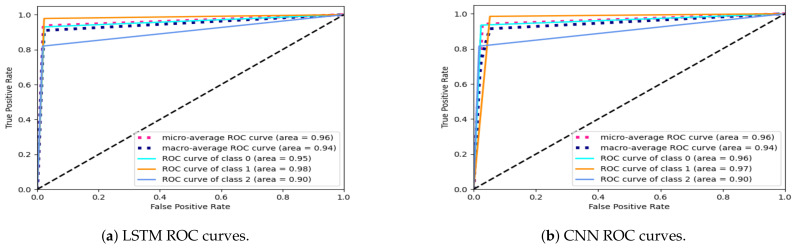
ROC curves computed for both classification frameworks, where class 0 corresponds to no activity, class 1 to walking, and class 2 to falling.

The other metric implemented to compare both classification frameworks was the ROC, whose graphs are shown in Figure 12. The graphs in Figure 12a,b show a low rate of FP for both classifiers. This behavior is expected in falling detection applications where the model’s performance needs to be maintained during the learning stage of the different classes of activities. The AUC for both schemes was 90%, which means that the quality of the predictions made by the algorithms is desirable.

The high accuracy values confirm that our fall detection system based on the analysis of Doppler signatures of CW RF signals generates enough information to sense these events. Furthermore, the platform uses a multi-class approach to distinguish different activities in a large indoor environment, with antennas located more than 7 m apart from each other. As has been addressed in this work, our principal concern is to detect falls as accurately as possible. The results show that our methodology is comparable to those proposed by other works based on classical radar concepts or CSI extraction. For example, in [12], the authors computed the CSI of WiFi signals, and with a CNN methodology, they obtained a percentage of 93.3% in fall detection. Works such as those presented in [9,33] used specialized high-resolution equipment such as Doppler radars to analyze the spectrograms of the signal. In this case, 95.00% and 95.60% precision were obtained in the fall class, respectively. Our work enables us to achieve a similar level of precision as these methodologies without the use of specialized equipment or jeopardizing the coexistence of joint radio and communications. Therefore, the concepts discussed here are proposed as a solution to some of the limitations of sensing systems based on RF signals.

## 6. Conclusions

In this work, we analyzed the performance of a fall detection platform based on CW RF signals using a multi-class DL framework. Using this platform, we collected data from probe signals containing Doppler signatures generated by people movement in an indoor environment. The activities of the experimentation protocol included walking, going up and down stairs, non-activity, and falls. Furthermore, we computed the spectrograms of each sequence of activities in the collected data to extract the features of the Doppler signatures. The spectrograms were divided into a training set and a validation and test set to be used in a DL framework. We compared the performance of two different multi-class classification approaches: an LSTM network and a CNN. The results showed an overall accuracy of 82.20% for the LSTM algorithm, with an precision of 70.50% in fall detection. On the other hand, the CNN reported an overall accuracy of 78.09% and a fall detection precision of 68.70%. We noted that elderly people have physical limitations regarding performing complex or highly mobile activities such as going up and down stairs. Therefore, we considered an experimental scenario where these kinds of activities are not carried out. We centered our efforts on sensing the activities of elderly people that live independently in one-story homes. In this way, the overall accuracy of the LSTM algorithm reached 94.95% and a precision of 92.1% in fall detection. Moreover, the CNN also increased in overall accuracy and reached 95.25%. In this case, the fall detection precision also achieved 92.1%. These results demonstrate that the features extracted from the Doppler signatures can be used in different classification frameworks with high levels of accuracy. Therefore, our platform could be used to monitor and detect falling events with a high accuracy rate. In addition, our methodology allows the integration of radio sensing and joint communications by using an RF probe signal similar to the pilot subcarriers of commercial WiFi systems. Nevertheless, the platform cannot be used to distinguish low-acceleration activities from the human body and is limited by these sensing capabilities. As future work, we will seek to increase the resolution of the captured Doppler signatures using other pre-processing techniques and a methodology to capture the signals with a better balance between acquisition time and frequency resolution.

## Figures and Tables

**Table 1:** Physical characteristics of the recruited participants.

Participant	Age	Gender	Height (m)	Weight (Kg)
1	21	Female	1.69	76
2	25	Female	1.57	50
3	26	Female	1.61	64
4	28	Female	1.64	59
5	47	Female	1.55	65
6	25	Male	1.67	70
7	29	Male	1.80	75
8	29	Male	1.63	93
9	30	Male	1.72	73
10	33	Male	1.65	72
11	48	Male	1.71	85

**Table 2:** Summary of trials performed by the participants.

Activity/Event	Trials per Participant	Total
Walk	30	330
Up/Down Stairs	10	110
Falls	10	110
No Activity	10	110

**Table 3:** Results of the metrics computed from the confusion matrices in the LSTM algorithm.

Metric	No Activity	Walking	Up/Down Stair	Falling
Precision	88.40%	89.90%	61.60%	70.50%
Recall	80.00%	97.60%	46.50%	76.50%
Specificity	97.51%	86.19%	93.88%	92.84%

**Table 4:** Results of the metrics computed from the confusion matrices in the CNN algorithm.

Metric	No Activity	Walking	Up/Down Stair	Falling
Precision	79.30%	86.20%	52.80%	68.70%
Recall	78.50%	98.60%	28.00%	68.70%
Specificity	95.03%	79.37%	94.65%	92.34%

## Data Availability

The data shown in this study are available upon reasonable request from the corresponding authors.
